# Combination of Antimalarial and CNS Drugs with Antineoplastic Agents in MCF-7 Breast and HT-29 Colon Cancer Cells: Biosafety Evaluation and Mechanism of Action

**DOI:** 10.3390/biom12101490

**Published:** 2022-10-16

**Authors:** Diana Duarte, Mariana Nunes, Sara Ricardo, Nuno Vale

**Affiliations:** 1 OncoPharma Research Group, Center for Health Technology and Services Research (CINTESIS), Rua Doutor Plácido da Costa, 4200-450 Porto, Portugal; 2 Faculty of Pharmacy, University of Porto, Rua Jorge Viterbo Ferreira, 228, 4050-313 Porto, Portugal; 3 CINTESIS@RISE, Faculty of Medicine, University of Porto, Alameda Professor Hernâni Monteiro, 4200-319 Porto, Portugal; 4 Differentiation and Cancer Group, Institute for Research and Innovation in Health (i3S), University of Porto/Institute of Molecular Pathology and Immunology, University of Porto (IPATIMUP), Rua Alfredo Allen 208, 4200-135 Porto, Portugal; 5 Institute of Biomedical Sciences Abel Salazar (ICBAS), University of Porto, Rua Jorge Viterbo Ferreira, 228, 4050-313 Porto, Portugal; 6 Toxicology Research Unit (TOXRUN), University Institute of Health Sciences, Polytechnic and University Cooperative (CESPU), Rua Central de Gandra, 1317, 4585-116 Gandra, Portugal; 7 Department of Community Medicine, Health Information and Decision (MEDCIDS), Faculty of Medicine, University of Porto, Rua Doutor Plácido da Costa, 4200-450 Porto, Portugal

**Keywords:** drug combination, drug repurposing, cancer therapy, CNS drugs, antimalarial drugs, PPT1

## Abstract

Drug combination and drug repurposing are two strategies that allow to find novel oncological therapies, in a faster and more economical process. In our previous studies, we developed a novel model of drug combination using antineoplastic and different repurposed drugs. We demonstrated the combinations of doxorubicin (DOX) + artesunate, DOX + chloroquine, paclitaxel (PTX) + fluoxetine, PTX + fluphenazine, and PTX + benztropine induce significant cytotoxicity in Michigan Cancer Foundation-7 (MCF-7) breast cancer cells. Furthermore, it was found that 5-FU + thioridazine and 5-fluorouracil (5-FU) + sertraline can synergistically induce a reduction in the viability of human colorectal adenocarcinoma cell line (HT-29). In this study, we aim to (1) evaluate the biosafety profile of these drug combinations for non-tumoral cells and (2) determine their mechanism of action in cancer cells. To do so, human fetal lung fibroblast cells (MRC-5) fibroblast cells were incubated for 48 h with all drugs, alone and in combination in concentrations of 0.25, 0.5, 1, 2, and 4 times their half-maximal inhibitory concentration (IC_50_). Cell morphology and viability were evaluated. Next, we designed and constructed a cell microarray to perform immunohistochemistry studies for the evaluation of palmitoyl-protein thioesterase 1 (PPT1), Ki67, cleaved-poly (ADP-ribose) polymerase (cleaved-PARP), multidrug resistance-associated protein 2 (MRP2), P-glycoprotein (P-gp), and nuclear factor-kappa-B (NF-kB) p65 expression. We demonstrate that these combinations are cytotoxic for cancer cells and safe for non-tumoral cells at lower concentrations. Furthermore, it is also demonstrated that PPT1 may have an important role in the mechanism of action of these combinations, as demonstrated by their ability to decrease PPT1 expression. These results support the use of antimalarial and central nervous system (CNS) drugs in combination regimens with chemotherapeutic agents; nevertheless, additional studies are recommended to further explore their complete mechanisms of action.

## 1. Introduction

According to the last report published by the American Cancer Society, it is estimated that more than 1,900,000 cancer cases will be diagnosed and about 600,000 cancer deaths will occur in the United States (US) in 2022 [1]. Along with lung cancer, breast and colorectal cancer (CRC) occupy the rank as the most diagnosed types of cancer, with an estimation of 43,780 cases and 52,580 estimated deaths in the US, in 2022 [1]. Although surgery and chemotherapy play a key role in cancer treatment, contributing to a positive outcome in many patients, chemotherapeutic drugs are not fully efficient and are accompanied by some toxicity, which can result in several side effects for the patients. Furthermore, it is common the development of tumor resistance to antineoplastic drugs, which contributes to a decrease in the efficacy rate of chemotherapy.

Surgery is the first line of treatment in early CRC, and it is usually accompanied by the use of chemotherapy. 5-fluorouracil (5-FU) is a chemotherapeutic drug used in the chemotherapy of CRC but its use is limited by its short half-life, low bioavailability, and severe side effects [2]. In the case of breast cancer, mastectomy is also one of the main strategies for tumor removal, but chemotherapy still plays an important role in the treatment. Paclitaxel (PTX) is an antineoplastic drug that acts as a mitotic inhibitor and is used for different types of cancer, including breast cancer. Nevertheless, its use is limited by the development of drug resistance and its undesired side effects [3]. Doxorubicin (DOX) is another chemotherapeutic drug commonly used for the treatment of breast cancer. Its mechanism of action involves the intercalation of this drug with the DNA chain, leading to the cleavage of DNA by the enzyme topoisomerase II and consequently stopping the cell cycle, resulting in cancer cell death [4]. The use of this drug is limited by its cardiotoxicity and the development of tumor resistance, which reduces the efficacy of the drug. Therefore, the development of novel, more efficient, and safer strategies for cancer treatment, that help increase the efficacy of the therapy as well as improve the quality of life of patients during their oncologic treatments, is currently a hot topic for the scientific community.

Two important strategies to overcome the aforementioned problems are drug repurposing and drug combination. Drug repurposing consists in finding novel indications for drugs that are already approved for clinical use [5]. Since these drugs have acceptable toxicological and pharmacokinetic profiles, widely described in the literature, their translation into other clinical indications results in a faster and more economical process [5]. Drug combination consists in using two or more drugs in the same therapeutic regimen [6], taking advantage of the different mechanisms of action of each drug to act in different pathways of tumorigenesis [7,8,9,10], helping surpass the resistance mechanisms of the tumor [11].

In our previous works [12,13], we developed a new combination model involving the use of antineoplastic drugs and repurposed drugs, aiming to improve the anticancer effects of chemotherapy in two different cancer cell lines: Michigan Cancer Foundation-7 (MCF-7,breast cancer) and human colorectal adenocarcinoma (HT-29, colorectal cancer) [12,13]. We screened several repurposed drugs [11 antimalarial and 17 central nervous system (CNS) drugs] [12,13], that were selected based on literature findings that suggested the repurposing potential of these drug classes. For that, we first incubated both cell lines with each repurposed drug alone, at increasing concentrations (0.1–100 µM), to find their half-maximal inhibitory concentration (IC_50_) value. Then, all repurposed drugs that presented an IC_50_ under 25 µM were considered to have a promising anticancer profile and therefore selected for the screening involving the drug combinations. Although the IC_50_ of chloroquine is above 25 µM, we also included this drug in the screening because approximately 30 clinical studies are currently evaluating the activity of this antimalarial drug combined with various standard treatments in different types of cancer. In breast cancer cells, the selected repurposed drugs were combined with two different antineoplastic drugs (PTX and DOX). In colon cancer cells, the selected repurposed drugs were only combined with 5-FU. We evaluated the cytotoxic effect of each drug pair by cell-based assays and evaluated the nature of drug interactions in each drug combination (additivity, synergism, or antagonism). Drug combinations that demonstrated the ability to synergistically enhance the chemotherapeutic effect of the antineoplastic drug were considered promising drug combinations for cancer therapy. The experiment protocol for drug combinations was selected based on Chou-Talalay publications [14,15], where it is recommended to use constant-ratio drug combinations and to select several data points above IC_50_ and several below IC_50_ to determine drug interactions more accurately.

We have found some combination regimens can effectively decrease the viability of these cell lines. Specifically, we have found the combinations of DOX + artesunate (ART), DOX + chloroquine (CQ), PTX + fluoxetine (FLUOX), PTX + fluphenazine (FLUPH), and PTX + benztropine (BENZ) to be the most cytotoxic for MCF-7 cells and to act synergistically. Regarding the results in HT-29 cells, we have found 5-FU + thioridazine (THIO) and 5-FU + sertraline (SERT) to be the drug combinations with the highest number of synergistic pairs [12,13]. 

We next determined the possible mechanisms of action of these drug combinations [16]. To do so, we evaluated the expression of several proteins related to epithelial-mesenchymal transition (EMT), e.g., E-cadherin, β-catenin, vimentin, and P-cadherin, and found an increase in E-cadherin expression together with a reduction in P-cadherin, vimentin, and β-catenin expression following drug combinations treatment, suggesting that these combinations can induce an EMT reversal [16]. Nevertheless, more studies are needed to further determine the mechanism of action of these combinations.

In this work, we propose two different objectives: (1) evaluate the biosafety of the previously mentioned drug combinations in a non-tumoral human fetal lung fibroblast cell line (MRC-5) and (2) determine the mechanisms of action of these combinations. First, we treated MRC-5 cells with each drug alone and in combination in the concentrations 0.25, 0.5, 1, 2, and 4 times the IC_50_ of each drug, and evaluated the cytotoxic effect of each treatment by MTT, after 48 h incubation. Then, we constructed a cell microarray (CMA) to evaluate the expression of several proteins in MCF-7 and HT-29 cells exposed to each treatment, by immunohistochemistry. The CMA is a single paraffin block that allows side-by-side comparations between different cell lines, diverse conditions, biomarker expression, and cytolocalization evaluation at the same time [17]. We evaluated the expression of the proteins palmitoyl-protein thioesterase 1 (PPT1), P-glycoprotein (P-gp), multidrug resistance-associated protein 2 (MRP2), Ki67, cleaved-poly (ADP-ribose) polymerase (cleaved-PARP) and nuclear factor-kappa-B (NF-kB) p65 after treatment with drugs, both alone and combined, in both cell lines. These proteins were studied for their implication in cancer: PPT1 is involved in lysosomal degradation and its high expression is correlated with a worse prognosis in cancer settings [18]. Several antimalarial drugs, such as CQ, are known to act by inhibiting this enzyme [18,19]. NF-kB p65 is a protein complex that regulates DNA transcription, cytokine production, and cell survival/death. Its inactive form is located in the cytoplasm and, upon activation, it migrates to the nucleus, enabling DNA transcription [20]. In cancer cells, a higher expression of NF-kB p65 is associated with a worse prognosis [20]. The P-gp, also known as MRP1, is a glycoprotein expressed in the cellular membrane and is involved in the efflux of drugs to the outside of cells [21]. Therefore, it has an important role in the development of drug resistance, being overexpressed in tumor cells [21]. The MRP2 protein is a member of the ABC transporters and has a similar function as P-gp [22]. Higher levels of MRP2 are related to chemoresistance [22]. The Ki67 is a marker for tumor cell proliferation; this protein is located in the nucleus and is involved in the regulation of mitosis [23]. This protein is used as a prognostic predictive biomarker in cancer patients, being an indicator of metastasis and tumor stage [23]. The PARP1 is a protein that is cleaved during apoptosis, being generated by the activation of caspases 3 and 7 [24]. This protein is involved in DNA repair and chromatin structure modulation. It is a nuclear protein and its levels are elevated in different types of cancer [24]. 

Taken together, the results present in this manuscript confirm the biosafety profiles of each compound and each combination to normal cells. Moreover, we demonstrate that PPT1 might be involved in the mechanism of action of the combinations evaluated in MCF-7 and HT-29 cells. Further studies are still necessary to determine the complete mechanisms of action behind these promising drug combinations involving antineoplastic and repurposed drugs. 

## 2. Materials and Methods

### 2.1. Biosafety Studies in MRC-5 Fibroblast Cells

#### 2.1.1. Cell Culture

MRC-5 human normal lung fibroblast cell line, obtained from the American Type Culture Collection (ATCC; Manassas, VA, USA), were used in passage 8 for evaluation of biosafety of the most promising drug combinations found in previous works [12,13]. These cells were maintained in Dulbecco’s modified Eagle’s medium (DMEM) cell culture medium supplemented with 10% fetal bovine serum (FBS) and 1% penicillin-streptomycin (pen-strep) solution, at 37 °C and 5% CO_2_. Cell culture reagents were purchased from Millipore Sigma (Merck KGaA, Darmstadt, Germany). For subculturing, confluent cells were trypsinized using a 0.25% trypsin-EDTA solution (Gibco, Thermo Fisher Scientific, Inc., Waltham, MA, USA) and subcultured in the same culture media. On the day before treatments, 8000 cells/well were seeded in 96-well plates and allowed to adhere for 24 h.

#### 2.1.2. Drug Treatment

Based on our previous studies [12,13], we selected the most promising drug combinations to evaluate their biosafety profile in normal cells. Previously, we have found that the combinations of DOX (cat. no. 15007, Cayman Chemical, Ann Arbor, MI, USA) + ART (cat. no. 11817, Cayman Chemical, Ann Arbor, MI, USA) and CQ (cat. no. C6628, Santa Cruz Biotechnology, Dallas, TX, USA), as well as PTX (cat. no. 1097, Tocris Bioscience, Bristol, UK) + FLUOX (cat. no. 14418, Cayman Chemical, Ann Arbor, MI, USA), FLUPH (cat. no. F4765, Merck KGaA, Darmstadt, Germany) and BENZ (cat. no. 16214, Cayman Chemical, Ann Arbor, MI, USA), resulted in significant cytotoxic effects in MCF-7 breast cancer cell line [12,13]. On the other hand, for HT-29 colon cancer cells, the most promising drug combinations included 5-FU (cat. no. F6627, Merck KGaA, Darmstadt, Germany) + THIO (cat. no. 14400, Cayman Chemical, Ann Arbor, MI, USA) and SERT (cat. no. 14839, Cayman Chemical, Ann Arbor, MI, USA) [12,13]. 

Based on these findings, the cytotoxic effect of different drug combinations (DOX + ART, DOX + CQ, PTX + FLUOX, PTX + FLUPH, PTX + BENZ, 5-FU + THIO and 5-FU + SERT) was evaluated in MRC-5 cells after 48 h of treatment. Cells were incubated first with each drug alone and then with the drug combinations. MRC-5 cells were incubated with the following concentrations: 0.25, 0.5, 1, 2, and 4 times the IC_50_ value of each drug. These IC_50_ values were based on our previous studies [12,13], where we found the IC_50_ for each drug in HT-29 and MCF-7 cells (Table 1). Here, we used the corresponding IC_50_ values in this cell line for comparison of the cytotoxicity. Stock solutions were prepared in 100% DMSO and diluted to 0.1% DMSO on the day of the experiments. Control cells were treated with vehicle (0.1% DMSO). No significant differences were seen between control cells with or without vehicle.

#### 2.1.3. Morphological Analysis

Morphological analysis was performed for all conditions using a Leica DMI 6000B microscope coupled with a Leica DFC350 FX camera (Leica Microsystems, Wetzlar, Germany). Images were treated with the Leica LAS X imaging software (v3.7.4) (Leica Microsystems, Wetzlar, Germany).

#### 2.1.4. MTT Assay

The cytotoxicity of each treatment was evaluated using the thiazolyl blue tetrazolium bromide (MTT) assay. Briefly, after cell treatment, each well aspirated and 100 µL of MTT solution (0.5 mg/mL in PBS, cat. no. M5655, Merck KGaA, Darmstadt, Germany) was added. Plates were protected from light and incubated for a period of 3 h at 37 °C. Then, the MTT solution was removed and replaced with 100 µL/well of DMSO to solubilize the formazan crystals. Absorbance was measured at 570 nm using an automated microplate reader (Tecan Infinite M200, Tecan Group Ltd., Männedorf, Switzerland).

#### 2.1.5. Selectivity Index (SI)

The SI of each drug alone was analyzed following the same methodology presented in [25,26] and was calculated as IC_50_ normal cell line/IC_50_ tumoral cell line. SI > 3 is indicative that the drug is selective, whereas SI > 10 indicates the drug is very selective. The SI evaluation was only performed for single-drug treatments as it is not possible to determine the IC_50_ of a drug combination. 

#### 2.1.6. Statistical Analysis

GraphPad Prism 9 (GraphPad Software Inc., San Diego, CA, USA) was used to obtain the cell viability representations. The results of three independent experiments are represented as the mean ± SEM. Statistical analysis was performed with one-way ANOVA tests by Dunnett’s multiple comparisons between control and treatment groups. Statistical significance was accepted at *p* values < 0.05.

### 2.2. Immunohistochemistry Studies in MCF-7 and HT-29 Cancer Cells

#### 2.2.1. Cell culture and Drug Treatment

Experiments were carried out using two cancer cell lines: MCF-7 breast and HT-29 colon (ATCC, American Type Culture Collection, Manassas, VA, USA). MCF-7 and HT-29 cancer cells were maintained in DMEM and McCoy’s (Merck KGaA, Darmstadt, Germany) cell culture medium, respectively, both supplemented with 10% FBS and 1% of a mixture of penicillin G and streptomycin (1000 U/mL; 10 mg/mL, Merck KGaA, Darmstadt, Germany). Cells were grown in T75 cm^2^ flasks and kept at 37 °C in a humidified atmosphere with 95% air and 5% CO_2_. Cell media was changed twice a week. When cell confluence reached 70–80% and for cell seeding prior experiences, cells were washed with phosphate-buffered saline (PBS, Gibco, Thermo Fisher Scientific, Inc., Waltham, MA, USA) and trypsinized using a solution of 0.25% trypsin-EDTA.

Before drug treatment, MCF-7 and HT-29 cells were plated in 6-well plates using a density of 7.5 × 10^5^ cells/well and incubated at 37 °C and 5% CO_2_ to adhere. After 24 h, cell media were replaced with drug-containing media for 48 h. For each condition, three wells were used. Based on the aforementioned results described in Section 2.1.2., MCF-7 cells were treated with vehicle (0.1% DMSO), PTX, DOX, ART, CQ, FLUOX, FLUPH, and BENZ alone and with the combinations of DOX + ART, DOX + CQ, PTX + FLUOX, PTX + FLUPHE and PTX + BENZ, at the concentration of 0.25 times their IC_50_ (Table 1). This concentration was selected so that we could have, simultaneously, some cytotoxic effect and an acceptable number of viable cells, to allow for marker quantification. HT-29 cells were treated with vehicle (0.1% DMSO), 5-FU, THIO, and SERT alone and with the combinations of 5-FU + THIO and 5-FU + SERT, at the concentration of 0.25 times their IC_50_ (Table 1). Cells were incubated with drugs alone or combined for 48 h. All drugs were dissolved in DMSO and kept at −20 °C until the experiments. No significant differences were seen between control cells with or without vehicle. 

#### 2.2.2. Collection, Fixation, and Histogel^TM^ Processing of Cultured Cells

After 48 h, cells were scraped from culture dishes, washed three times with ice-cold PBS and fixed with 10% (*v/v*) neutral-buffered formaldehyde (AppliChem GmbH, Darmstadt, Germany), and fixed for 1 h at RT with gentle agitation. Then, the cells’ pellet was re-suspended in liquefied HistoGel™ (Thermo Fisher Scientific, Inc., Waltham, MA, USA), according to the manufacturer’s instructions, followed by standard histological processing and paraffin embedding. To confirm that all conditions tested were appropriate for the CMA construction, each block was sectioned with a microtome, and hematoxylin and eosin (H&E) staining was performed.

#### 2.2.3. CMA Construction and Immunocytochemistry Expression Analysis

All conditions were arrayed in a CMA block, in the same way as previously described by Nunes et al. [27]. After designing the cell block, it was constructed and finally sectioned using a microtome. For the immunocytochemistry protocol, the slides were deparaffinized and hydrated. Next, the antigen retrieval (unmasking) was performed by heat-induction (98 °C), using a citrate buffer solution (1:100 at pH 6.0; Thermo Fisher Scientific, Inc., Waltham, MA, USA) or ethylenediamine tetraacetic acid (EDTA; 1:100; Thermo Fisher Scientific, Inc., Waltham, MA, USA) for 40 min. Then, the activity of endogenous peroxidase was blocked using a hydrogen peroxide solution 3% (*v/v*) (Thermo Fisher Scientific, Inc., Waltham, MA, USA) for 10 min. Finally, each slide was incubated with the specific primary antibodies (conditions described in Supplementary Table S1). Then, was detected using a secondary antibody with horseradish peroxidase (HRP)-labeled polymer (Dako REAL™ EnVision™ Detection System Peroxidase/DAB+, Rabbit/Mouse) for 30 min. Diaminobenzidine was used to develop the activity of peroxidase according to the manufacturer’s instructions. Hematoxylin staining was also performed to stain the nucleus of the cells. In the last step, slides were dehydrated, clarified, and coverslipped using a mounting medium for microscopic visualization and analysis. The staining pattern (nuclear, cytoplasm, or membrane) and the percentage of cells stained (0%, 1–10%, 11–25%, 26–50%, 51–75%, and 76–100%) were evaluated and validated by three independent observers (DD, MN, and SR).

## 3. Results and Discussion

### 3.1. Biosafety Studies in MRC-5 Fibroblast Cells

#### 3.1.1. Single Drug Treatments

In our previous studies, we developed a novel model of combination consisting of antineoplastic and repurposed drugs [12,13]. These studies aimed to find repurposed drugs with a promising anticancer activity that could, at the same time, be used in combination with antineoplastic drugs to improve their cytotoxicity against two different cancer cell lines: MCF-7 and HT-29 cancer cells. In these experiments, we first determined the IC_50_ of every single drug and then selected the ones with the most promising pharmacological profiles (defined as an IC_50_ < 25 µM, Table 1) [12,13]. Although the IC_50_ of chloroquine is above 25 µM, we also included this drug in the screening because approximately 30 clinical studies are currently evaluating the activity of this antimalarial drug combined with various standard treatments in different types of cancer. Next, we combined simultaneously the antineoplastic drugs with the selected repurposed drugs in variable concentrations and evaluated the combined effects of equipotent concentrations (fixed ratio) of the IC_50_ values for each drug (0.25, 0.5, 1, 2, and 4 times IC_50_). This experiment protocol for drug combinations was selected based on Chou-Talalay publications [14,15], where it is recommended to use constant-ratio drug combinations and to select several data points above IC_50_ and several below IC_50_ to determine drug interactions more accurately. We successfully identified several antimalarial and CNS drugs with potential anticancer activity and demonstrated that the most promising drug combinations were DOX + ART, DOX + CQ, PTX + FLUOX, PTX + FLUPH, PTX + BENZ for MCF-7 cells, and 5-FU + THIO and 5-FU + SERT for HT-29 cells [12,13]. 

Here, we investigated if these drug combinations are safe against non-tumoral cells. First, to evaluate whether the antineoplastic drugs used in the previously proposed combinations are selective for cancer cells and not cytotoxic for normal cells, we evaluated each drug, both alone and in combination in MRC-5, a human normal lung fibroblast cell line. This cell line is commonly used in the production of vaccines, for biosafety evaluation. To do so, we evaluated the cytotoxic effect of each antineoplastic drug, in a range of concentrations from 0.25 to 4 times their IC_50_ value for 48 h. Cell viability was assessed using an MTT assay. Drugs that did not cause significant reduction in the viability of MRC-5 cells were considered to have an acceptable toxicological profile and to be non-toxic for normal cells [29].

**Figure 1:**
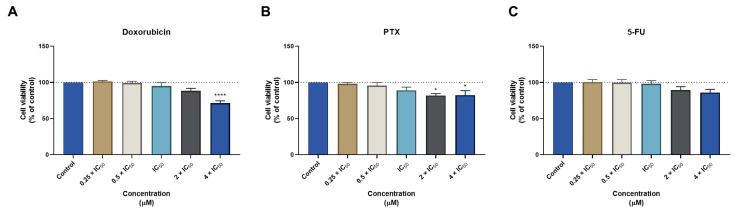
Cytotoxic effects of antineoplastic drugs in human fetal lung fibroblast cell line (MRC-5). MTT results of MRC-5 cells treated with increasing concentrations of (**A**) doxorubicin (DOX), (**B**) paclitaxel (PTX), and (**C**) 5-fluorouracil (5-FU) for 48 h. Results are represented as the percentage of control and represent means ± SEM. Each experiment was done three times independently (*n* = 3). Statistically significant vs control at *p* < 0.05 * and *p* < 0.0001 ****.

In our previous studies using tumoral cell lines, we found an IC_50_ of 0.17 µM and 0.44 nM for DOX and PTX, respectively, in MCF-7 cells and 3.79 µM for 5-FU in HT-29 cancer cells. Here, using a non-tumoral cell line, we demonstrate that all drugs present an acceptable toxicological profile for all chemotherapeutic agents, at all concentrations under their IC_50_. Significant toxicity was only found in the treatment with DOX in the concentration of 4 × IC_50_ (Figure 1A). Furthermore, treatment with 2 and 4 × IC_50_ of PTX also resulted in significant cytotoxicity for MRC-5 cells (Figure 1B). On the other hand, results regarding treatment with 5-FU did not demonstrate significant cytotoxicity at any concentration (Figure 1C). Together, these results demonstrate that DOX, PTX, and 5-FU are non-toxic for normal cells and support their use as anticancer agents. Regarding selectivity, we found these antineoplastic drugs are not selective for cancer cells (Table 2).

**Figure 2:**
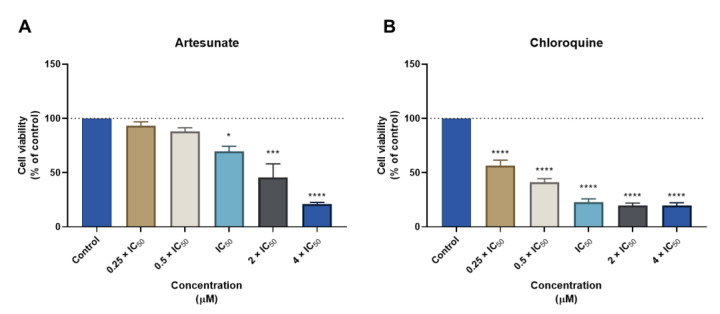
Cytotoxic effects of antimalarial drugs in MRC-5 human normal lung fibroblast cells. MTT results of MRC-5 cells treated with increasing concentrations of (**A**) artesunate (ART) and (**B**) chloroquine (CQ) for 48 h. Results are represented as the percentage of control and represent means ± SEM. Each experiment was done three times independently (*n* = 3). Statistically significant vs. control at *p* < 0.05 *, *p* < 0.001 *** and *p* < 0.0001 ****.

Next, we evaluated the cytotoxic effect of the antimalarial drugs used in the previously mentioned drug combinations. We found these drugs are more cytotoxic for MCR-5 cells than the antineoplastic drugs DOX, PTX, and 5-FU. ART treatment induced a significant reduction in cell viability in concentrations of IC_50_ and higher (Figure 2A). On the other side, CQ treatment demonstrated toxicity for all the ranges of concentrations tested for this cell line (Figure 2B). This demonstrates that, although antimalarial drugs alone can be promising anticancer agents, they also induce cytotoxicity to normal cells, which warns their use as standalone therapeutics for cancer therapy. Furthermore, we demonstrated these antimalarial drugs are not selective against cancer cells (Table 3).

**Figure 3:**
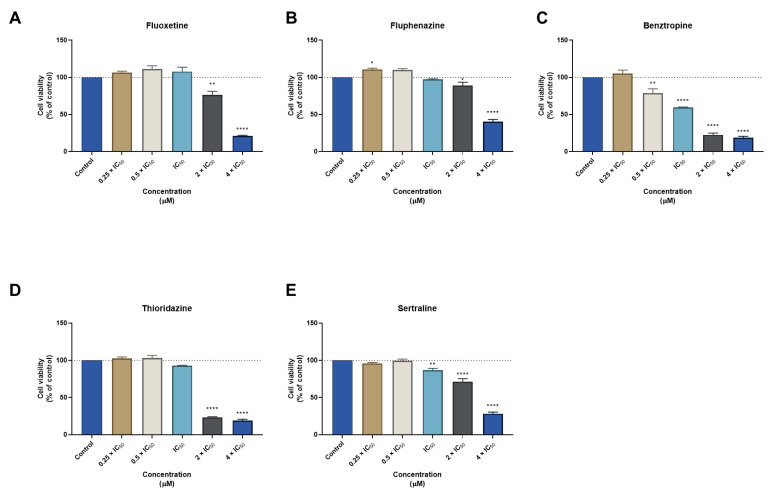
Cytotoxic effects of CNS drugs in MRC-5 human normal lung fibroblast cells. MTT results of MRC-5 cells treated with increasing concentrations of (**A**) fluoxetine (FLUOX), (**B**) fluphenazine (FLUPH), (**C**) benztropine (BENZ), (**D**) thioridazine (THIO), and (**E**) sertraline (SERT) for 48 h. Results are represented as the percentage of control and represent means ± SEM. Each experiment was done three times independently (*n* = 3). Statistically significant vs. control at *p* < 0.05 *, *p* < 0.01 ** and *p* < 0.0001 ****.

Finally, the biosafety of CNS drugs was also evaluated on MRC-5 cells. MTT results demonstrated that FLUOX treatment did not induce cell cytotoxicity in concentrations under IC_50_ (Figure 3A). Moreover, treatment with FLUPH resulted in a similar toxicological profile to FLUOX, with an acceptable biosafety profile to MRC-5 cells at the concentrations of 0.25, 0.5, and 1 times IC_50_ (Figure 3B). On the other hand, BENZ treatment demonstrated a compatible cytotoxic profile to normal cells only at the lowest concentration (0.25 × IC_50_) (Figure 3C). Indeed, BENZ was the most cytotoxic compound among all CNS drugs tested. THIO treatment only induced significant reductions of cell viability in concentrations above IC_50_, without significant cytotoxicity at the concentrations of 0.25, 0.5, and 1 times IC_50_ (Figure 3D). SERT, along with BENZ, was the second most cytotoxic compound for MRC-5 cells, with an acceptable biosafety profile only at the concentration of 0.5 × IC_50_ (Figure 3E). These results demonstrate that the selected CNS drugs have an acceptable toxicological profile at lower doses, supporting their use as standalone or adjuvant compounds for cancer therapy. Nevertheless, it was demonstrated these CNS drugs are not selective against cancer cells (Table 4).

Supplementary Figure S1 summarizes the previously described results. Taken together, these results demonstrate that antineoplastic drugs are safe for normal cells at low concentrations, with an acceptable toxicological profile. Moreover, we showed that antimalarial drugs are more cytotoxic than CNS drugs in MRC-5 human normal lung fibroblast cells, with CQ causing significant cell reduction even at lower concentrations. The selected CNS drugs only produced significant reductions in cell viability at higher concentrations. Nevertheless, it was demonstrated that these drugs are not selective against cancer cells.

#### 3.1.2. Combination Treatments

Next, we evaluated the safety of the drug combinations in MCR-5 cells. MRC-5 cells were treated with the antineoplastic and repurposed drugs simultaneously, using a fixed ratio of concentrations: 0.25, 0.5, 1, 2, and 4 times the IC_50_ value of each drug. Cell morphology and viability were evaluated after 48 h incubation.

**Figure 4:**
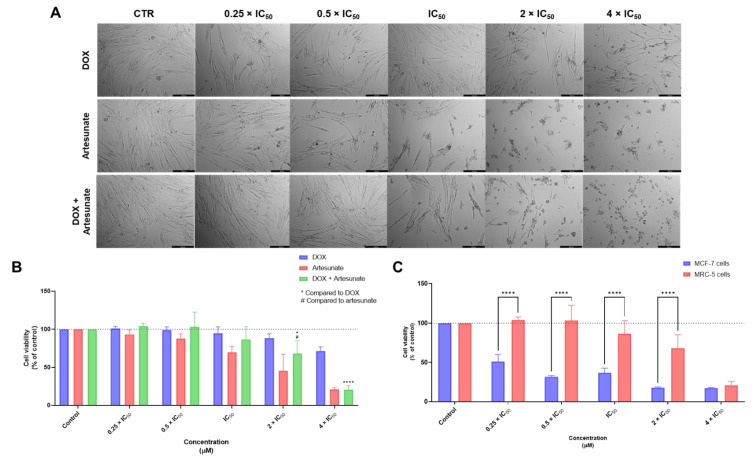
Cytotoxic effects of DOX + ART in MRC-5 human normal lung fibroblast cells. (**A**) Morphological analysis and (**B**) MTT results of MRC-5 cells treated with increasing concentrations of DOX, ART, and DOX + ART for 48 h. (**C**) Comparison of cytotoxicity of combined treatment with DOX + ART for 48 h in MCF-7 breast cancer cells and MRC-5 normal cells. Results are represented as the percentage of control and represent means ± SEM. Each experiment was done three times independently (*n* = 3). Statistically significant at *p* < 0.05 * and *p* < 0.0001 ****. Statistically significant compared to ART at *p* < 0.05 ^#^. Scale bar 200 µm.

Although some repurposed drugs demonstrated cytotoxicity against normal cells as single agents, their biosafety profile was further evaluated in combination treatments to find if the combination of these drugs with antineoplastic agents could result in lower toxicity for normal cells. Regarding the combination of DOX + ART, we have found changes in the normal phenotype of MRC-5 cells treated with DOX + ART in concentrations above IC_50_ (Figure 4A). The MTT results demonstrated this drug combination only presented significant cytotoxicity in concentrations above IC_50_ (Figure 4B). Furthermore, the combination of these drugs presented less cytotoxicity than each drug alone in concentrations of 0.25 and 0.5 times IC_50_, demonstrating that the association of ART with DOX can effectively decrease the toxicity of each drug. Moreover, it was found that, in some conditions (IC_50_ and 2 × IC_50_), the toxicity of ART was reduced when combined with DOX, which was also supported by morphological analysis. Furthermore, compared to the results previously obtained in MCF-7, it was notorious the biosafety profile of this drug combination in MRC-5 cells and the selectivity of DOX + ART to cancer cells (Figure 4C).

**Figure 5:**
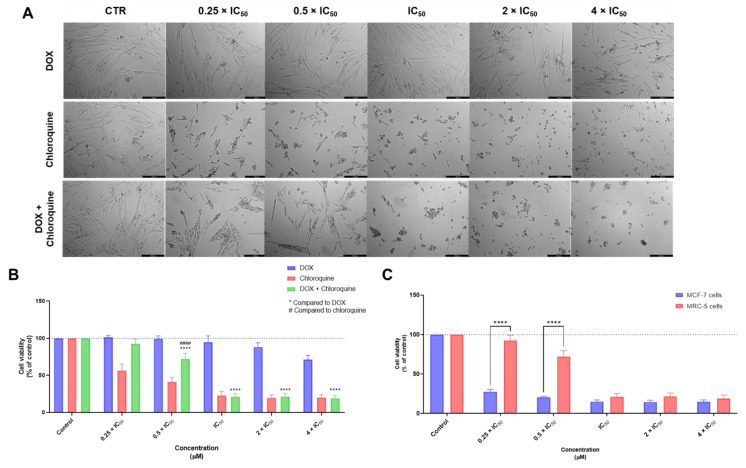
Cytotoxic effects of DOX + CQ in MRC-5 human normal lung fibroblast cells. (**A**) Morphological analysis and (**B**) MTT results of MRC-5 cells treated with increasing concentrations of DOX, CQ, and DOX + CQ for 48 h. (**C**) Comparison of cytotoxicity of combined treatment with DOX + CQ for 48 h in MCF-7 breast cancer cells and MRC-5 normal cells. Results are represented as the percentage of control and represent means ± SEM. Each experiment was done three times independently (*n* = 3). Statistically significant at *p* < 0.0001 ****. Statistically significant compared to CQ at *p* < 0.0001 ^####^. Scale bar 200 µm.

As expected, and in line with the results obtained for single drug treatments, the combination of DOX + CQ resulted in higher cytotoxicity to MRC-5 cells than DOX + ART. The morphological analysis supported the toxic profile of the combination, with notorious changes in the cell morphology, even for the lowest concentrations (Figure 5A). Specifically, the combination of 0.25 × IC_50_ of DOX + 0.25 × IC_50_ of CQ did not produce significant cytotoxic effects in these normal cells (Figure 5B). Compared to the results in MCF-7, this combination seems to be safe in the concentrations of 0.25 and 0.5 times IC_50_, while having potent anticancer activity against tumor cells (Figure 5C) and further supporting the study of this combination. 

**Figure 6:**
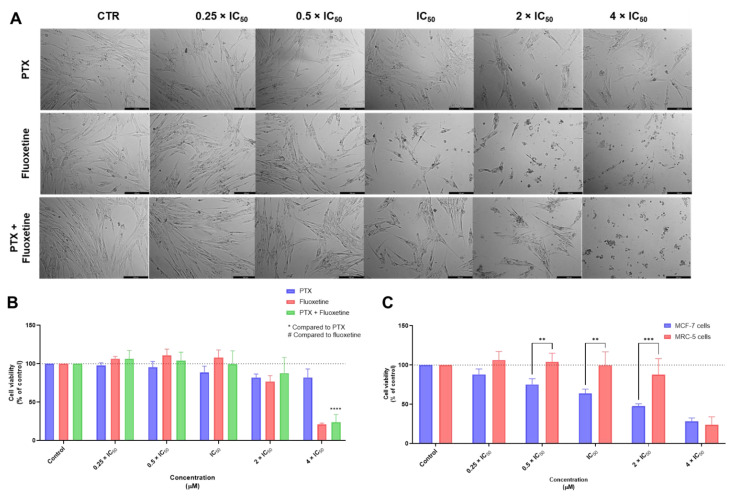
Cytotoxic effects of PTX + FLUOX in MRC-5 human normal lung fibroblast cells. (**A**) Morphological analysis and (**B**) MTT results of MRC-5 cells treated with increasing concentrations of PTX, FLUOX, and PTX + FLUOX for 48 h. (**C**) Comparison of cytotoxicity of combined treatment with PTX + FLUOX for 48 h in MCF-7 breast cancer cells and MRC-5 normal cells. Results are represented as the percentage of control and represent means ± SEM. Each experiment was done three times independently (*n* = 3). Statistically significant at *p* < 0.01 **, *p* < 0.001 *** and *p* < 0.0001 ****. Scale bar 200 µm.

The combination of PTX + FLUOX did not produce significant alterations in cell viability at lower concentrations, which is supported by the microscopic images of morphological analysis (Figure 6A). The MTT results regarding the combination of PTX + FLUOX revealed a promising safety profile of this combination, for all concentrations, except for 4 × IC_50_ (Figure 6B). Furthermore, compared to MCF-7 cells, we demonstrated this drug is safe against MRC-5 cells, at all concentrations from 0.25 to 2 × IC_50,_ and to be cytotoxic for cancer cells (Figure 6C). These results demonstrate that FLUOX can be effectively used in combination with PTX, with a safety profile against normal cells and high efficacy against cancer cells. 

**Figure 7:**
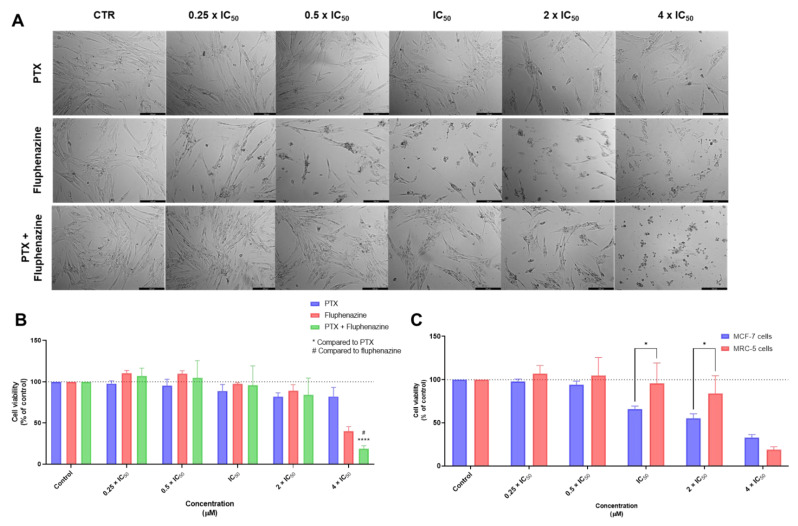
Cytotoxic effects of PTX + FLUPH in MRC-5 human normal lung fibroblast cells. (**A**) Morphological analysis and (**B**) MTT results of MRC-5 cells treated with increasing concentrations of PTX, FLUPH, and PTX + FLUPH for 48 h. (**C**) Comparison of cytotoxicity of combined treatment with PTX + FLUPH for 48 h in MCF-7 breast cancer cells and MRC-5 normal cells. Results are represented as the percentage of control and represent means ± SEM. Each experiment was done three times independently (*n* = 3). Statistically significant at *p* < 0.05 * and *p* < 0.0001 ****. Statistically significant compared to FLUPH at *p* < 0.05 ^#^. Scale bar 200 µm.

Next, the cytotoxic effect of the combination of PTX + FLUPH was further evaluated in MRC-5 cells. The results of cellular morphology analysis were in accordance with the MTT results, with significant changes in the phenotype of MRC-5 cells in the concentration of 4 times IC_50_ (Figure 7A). Analyzing the MTT results, it was possible to confirm this combination regimen is safe for this cell line in the concentrations of 0.25, 0.5, 1, and 2 times IC_50_ (Figure 7B). Moreover, the comparison of the cytotoxic effect between tumoral MCF-7 cells and non-tumoral MRC-5 cells demonstrated that, at lower concentrations, this combination has a promising toxicological profile, being safe for normal cells and effective against tumor cells (Figure 7C). 

**Figure 8:**
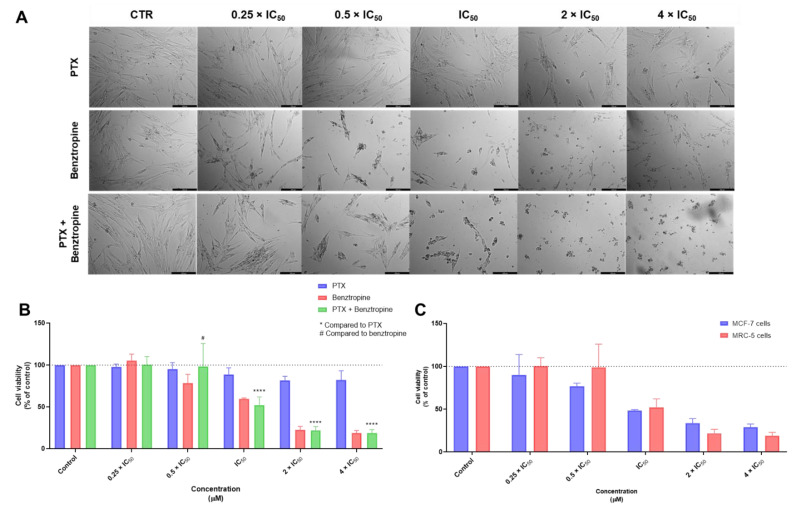
Cytotoxic effects of PTX + BENZ in MRC-5 human normal lung fibroblast cells. (**A**) Morphological analysis and (**B**) MTT results of MRC-5 cells treated with increasing concentrations of PTX, BENZ, and PTX + BENZ for 48 h. (**C**) Comparison of cytotoxicity of combined treatment with PTX + BENZ for 48 h in MCF-7 breast cancer cells and MRC-5 normal cells. Results are represented as the percentage of control and represent means ± SEM. Each experiment was done three times independently (*n* = 3). Statistically significant at *p* < 0.0001 ****. Statistically significant compared to BENZ at *p* < 0.05 ^#^. Scale bar 200 µm.

Regarding the combination of PTX plus BENZ, it was found this combination to be more cytotoxic to MRC-5 cells than PTX + FLUOX and PTX + FLUPH, which was also in line with the previous results obtained for single drug treatments. The morphological analysis demonstrated a reduction of cell viability and changes in cell morphology in the concentration of 0.5 × IC_50_ (Figure 8A), warning the use of this combination. Nevertheless, this combination demonstrated to have an acceptable toxicological profile at concentrations of 0.25 and 0.5 times IC_50_ (Figure 8B). The results of PTX + BENZ in MRC-5 and MCF-7 cells support the use of this combination at lower concentrations in cancer therapy (Figure 8C). 

**Figure 9:**
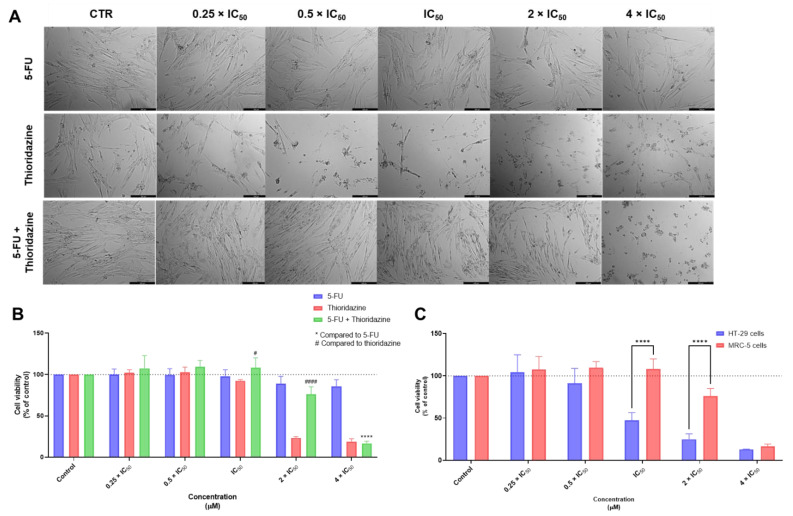
Cytotoxic effects of 5-FU + THIO in MRC-5 human normal lung fibroblast cells. (**A**) Morphological analysis and (**B**) MTT results of MRC-5 cells treated with increasing concentrations of 5-FU, THIO, and 5-FU + THIO for 48 h. (**C**) Comparison of cytotoxicity of combined treatment with 5-FU + THIO for 48 h in HT-29 colon cancer cells and MRC-5 normal cells. Results are represented as the percentage of control and represent means ± SEM. Each experiment was done three times independently (*n* = 3). Statistically significant at *p* < 0.0001 ****. Statistically significant compared to THIO at *p* < 0.05 ^#^ and *p* < 0.0001 ^####^. Scale bar 200 µm.

The combination of the antineoplastic drug 5-FU with THIO did not demonstrate significant changes in cell phenotype in concentrations under 2 × IC_50_ (Figure 9A). Regarding the MTT results, this combination demonstrated low cytotoxicity against MRC-5 cells in concentrations below the IC_50_, with no significant changes in cell viability in this range of concentrations (Figure 9B). Comparing the cytotoxic effects of this combination between tumoral and non-tumoral cells (Figure 9C), it was found this drug significantly reduced the cell viability of HT-29 colon cancer, without affecting MRC-5 fibroblast cells. These results support the use of this combination in cancer therapies. 

**Figure 10:**
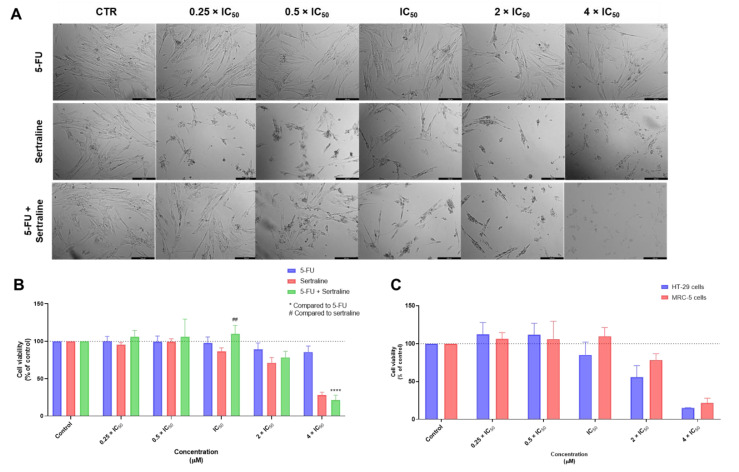
Cytotoxic effects of 5-FU + SERT in MRC-5 human normal lung fibroblast cells. (**A**) Morphological analysis and (**B**) MTT results of MRC-5 cells treated with increasing concentrations of 5-FU, SERT, and 5-FU + SERT for 48 h. (**C**) Comparison of cytotoxicity of combined treatment with 5-FU + SERT for 48 h in HT-29 colon cancer cells and MRC-5 normal cells. Results are represented as the percentage of control and represent means ± SEM. Each experiment was done three times independently (*n* = 3). Statistically significant at *p* < 0.0001 ****. Statistically significant compared to SERT at *p* < 0.01 ^##^. Scale bar 200 µm.

Finally, we evaluated the cytotoxic effect of 5-FU combined with SERT in MRC-5 cells. The results of the morphological analysis demonstrated some degree of cytotoxicity even in concentrations of 0.5 × IC_50_, with a decrease in the cell number as well as changes in the cell phenotype (Figure 10A). Nevertheless, the MTT assay demonstrated this combination did not induce significant effects on the cell viability of this cell line, in concentrations below and equal to IC_50_ (Figure 10B). It is, therefore, advised to use this drug combination at lower concentrations (0.25 × IC_50_). The results described in Figure 10C demonstrated that in the range of concentrations this combination is safe (0.25–1 × IC_50_), there was almost no anticancer effect against HT-29 colon cancer cells, which suggests that the use of this drug combination in cancer therapy may not be fully adequate. 

Figure S2 summarizes the previously described results. Taken together, these results are in line with the ones obtained for MRC-5 treated with single drugs. DOX + CQ is among the most cytotoxic drug combinations against this cell line and its use is only adequate at lower concentrations. PTX + BENZ is the second most cytotoxic drug combination and its use in cancer therapies is only possible in concentrations below 0.5 × IC_50_. The remaining drug combinations present selectivity for tumoral cells and are safe for normal cells, especially at concentrations till their IC_50_. At the concentration of 4 times IC_50_, all tested drug pairs have significant cytotoxicity against MRC-5 normal cells. We also demonstrate that these combinations help to reduce the cytotoxicity of the repurposed drugs alone. These results support the use of these drug combinations in further studies, using animal models, although the use of lower doses is recommended.

### 3.2. Immunohistochemistry Studies in MCF-7 and HT-29 Cancer Cells

After proving that the proposed drug combinations have an acceptable toxicological profile against non-tumoral cancer cells while retaining significant anticancer effects against different tumoral cancer cells, we next aimed to evaluate the expression of different proteins that are related to carcinogenesis. To do so, we cultured MCF-7 breast cancer cells and treated them with the corresponding drug combinations: DOX + ART, DOX + CQ, PTX + FLUOX, PTX + FLUPH, and PTX + BENZ for 48 h. HT-29 cells were treated with the combinations of 5-FU plus THIO and SERT. Cells were treated with a concentration of 0.25 × IC_50_ of each drug, to ensure enough viable cells for the IHC evaluation. A CMA composed of positive controls and each condition was constructed and used to perform IHC evaluation using the following antibodies: MRP2, P-gp, Ki67, NF-kB p65, cleaved-PARP, and PPT1. Figure S3 represents the IHC results of a liver used as a positive control for PPT1, MRP2, and P-gp. As expected, it was found an abundant expression of PPT1 in the cytoplasm of liver cells (Figure S3A), as this enzyme is involved in lysosomal degradation. On the other side, regarding P-gp (Figure S3B) and MRP-2 (Figure S3C), since these proteins are involved in the efflux of drugs to the outside of cells, it was found an abundant expression in the cell membrane. These results support the selectivity of these antibodies and demonstrate that the IHC protocol has been correctly performed. 

#### 3.2.1. MCF-7 Cells Treated with DOX + Antimalarial Drugs

First, we evaluated the expression of Ki67, cleaved-PARP, and NF-kB p65 in MCF-7 cells treated with a combination of DOX plus the antimalarial drugs ART and CQ (Figure S4).

Ki67 is a nuclear protein that marks active cell proliferation, both in normal and tumoral cells [30]. Specifically, this protein is used as a marker for the classification, prognosis, and prediction of therapeutic response in breast cancer [30]. This protein is expressed in all active phases of the cell cycle and decreases its expression after mitosis, in G0 phase [31]. Here, we hypothesize if the drug combinations could effectively reduce cell proliferation and consequently the expression of Ki67. As expected, in the control condition was found abundant nuclear expression (51–75%) of this protein in MCF-7 cells (Figure S4). On the other side, MCF-7 treated with DOX alone demonstrated a decreased expression of Ki67 (26–50%) when compared to control (Figure S4). Nevertheless, treatment with antimalarial drugs alone did not cause a decrease in MCF-7 cell proliferation; treatment with ART alone demonstrated a similar expression of Ki67 to control cells, and treatment with CQ alone caused an elevated expression of Ki67 (76–100%) compared to control cells (51–75%) (Figure S4). The combination treatment of DOX + ART resulted in similar results to control cells regarding Ki67 expression (51–75%) (Figure S4). Interestingly, when DOX was combined with CQ it was found a decrease in Ki67 expression (26–50%), both compared to control cells and to the treatment with CQ alone, which demonstrates this combination can reduce MCF-7 cell proliferation (Figure S4). 

PARP is a protein involved in DNA repair and chromatin modulation, being overexpressed in cancer [32]. Therefore, PARP is an important target for cancer therapy. Here, we hypothesize if these drug combinations can act as PARP inhibitors. It was not found significant changes in cleaved-PARP expression among treatments with single and combined drugs, with all cleaved-PARP expression results being negative or <1% (Figure S4). NF-kB is a well-known signaling pathway in oncogenesis and aberrant activation of this pathway is already described for different types of cancer [33]. The results found with respect to NF-kB p65 expression were similar to those regarding PARP expression, which indicates these combinations do not target the NF-kB pathway (Figure S4). 

**Figure 11:**
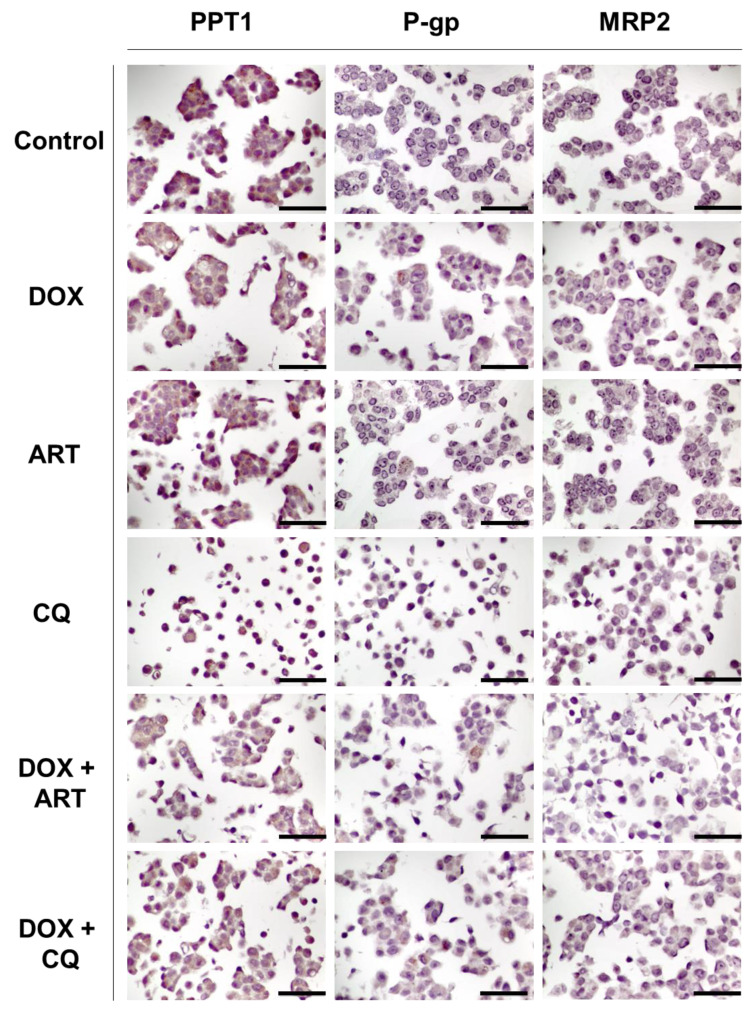
Representative IHC images for palmitoyl-protein thioesterase 1 (PPT1), P-glycoprotein (P-gp), and multidrug resistance-associated protein 2 (MRP2) expression in MCF-7 cells treated with antimalarial drugs (ART and CQ), alone and in combination with DOX. All images are taken at magnification of 400×. Scale bar 50 µm.

Next, we evaluated the expression of PPT1, P-gp, and MRP-2 in MCF-7 cells treated with a combination of DOX plus the antimalarial drugs ART and CQ (Figure 11). 

PPT1 is an enzyme involved in lysosomal degradation and is overexpression in different types of cancer. CQ is known to inhibit this enzyme [18,19]. The activity of PPT1 in cancer cells involves the depalmitoylation of the V0a1 subunit of v-ATPase, which facilitates the v-ATPase assembly on the lysosomal membrane [34]. Then, the V1 subunit of v-ATPase interacts with Ragulator, enabling the formation of the mTORC1 complex on the lysosome and activating mTORC1. This activation induces protein synthesis and inhibits the autophagic process [34]. PPT1 can also remove palmitate from palmitoylated proteins prior to their degradation in the lysosome. PPT1 inhibition impairs the depalmitoylation of the V0a1 subunit of v-ATPase, disrupting the assembly of v-ATPase and causing lysosomal deacidification [34]. Furthermore, the interaction between the V0a1 subunit of v-ATPase and Ragulator is lost, causing the inhibition of mTORC1, which inhibits protein synthesis and stops cell proliferation and tumor growth, and impairs autophagy [34]. In this work, we hypothesize if the proposed drug combinations can inhibit PPT1 enzyme. In MCF-7 control cells, it was found low expression (11–25%) of PPT1 (Figure 11). Treatment with DOX and ART alone led to an increase in PPT1 expression to 26–50%, while treatment with CQ resulted in 75–100% of MCF-7 expressing this protein, contrary to what was expected (Figure 11). Results of MCF-7 cells treated with DOX + ART demonstrated a similar expression of PPT1 to DOX and ART alone (Figure 11). Nevertheless, results regarding the combination of DOX + CQ resulted in a significant decrease in PPT1 expression (11–25%), demonstrating the ability of the combination to inhibit PPT1 expression (Figure 11). 

P-gp, also known as multidrug resistance protein (MDR1), is an adenosine triphosphate (ATP) binding cassette transporter (ABCB1) that is expressed in the cell membrane and is involved in the efflux of drugs to the outside of cells, contributing to the development of drug resistance [35]. Many types of cancer overexpress Pgp which prevents cancer drugs from reaching their cellular targets [36]. Antineoplastic drugs that interfere with the DNA replication process, such as doxorubicin, are especially vulnerable to P-gp overactivity [37]. On the other hand, novel drugs that inhibit P-gp can be promising agents for cancer cell growth inhibition and overcoming multidrug resistance [37]. Ras, c-Raf, p53, and other oncogenes are reported to affect the regulation of P-gp expression [38]. Moreover, it is described that epigenetic modifications play a role in the activation of P-gp-mediated drug resistance [39]. C/EBPβ has also been shown to induce P-gp expression in MCF-7 breast cancer cells. Furthermore, it has been demonstrated that hypertonic and hypoxic stress increases P-gp expression [40,41]. Here, we hypothesize these drug combinations can effectively inhibit P-gp activity, contributing to retaining drugs inside cells. Control cells did not demonstrate any expression of this protein. Treatment with DOX resulted in an increased expression of P-gp (11–25%) (Figure 11). Interestingly, we have found marking of this protein in the cytoplasm in all conditions, although this protein is usually located in the cell membrane. We could not find any explanation for this event, as there are no studies that describe the migration of this protein to the cytoplasm. Treatment with ART did not change PPT1 expression compared to control but the combination of DOX + ART resulted in higher expression of this protein (11–25%) (Figure 11). On the other side, MCF-7 cells treated with CQ alone and combined with DOX revealed a higher expression of PPT1 (26–50%) (Figure 11). 

The MRP2 protein is a member of the ABC transporters and has a similar function as P-gp. MRP2 is expressed in various tissues such as hepatocytes, kidney proximal tubules, intestine, nerves, bladder, placenta, and CD4+ lymphocytes [42]. In vivo studies using MRP2−/− mice demonstrated that MRP2 knockdown decreases hepatobiliary excretion of drugs and toxins as well as their metabolites, suggesting that MRP2 may play a role in eliminating endogenous and xenobiotic metabolites [43]. Moreover, additional studies have demonstrated that SNPs greatly influence the function of MRP2, such as substrate effluxing and miRNA recognition [44]. Several studies implicate MRP2 in the chemoresistance of some types of cancer [45]. Indeed, in tumor cells, it was found that MRP2 mRNA levels are related to resistance to chemotherapeutic drugs such as vincristine, cisplatin, and doxorubicin [46]. We hypothesize if the combination pairs can target MRP2 and inhibit this protein. Results regarding MRP-2 expression demonstrated this protein is not expressed in these cells in any treatment (Figure 11). 

#### 3.2.2. MCF-7 Cells Treated with PTX + CNS Drugs

Then, we evaluated the expression of Ki67, cleaved-PARP, and NF-kB p65 in MCF-7 cells treated with a combination of PTX plus the CNS drugs FLUOX, FLUPH, and BENZ (Figure S5). It was found an abundant expression (51–75%) of Ki67 in the nucleus of MCF-7 control cells. On the other side, all treatments with PTX and CNS drugs, both alone and combined, presented higher Ki67 expression (76–100%) (Figure S5). It was not found significant changes in cleaved-PARP and NF-kB p65 expression among treatments with single and combined drugs, with a negative or <1% expression (Figure S5). 

**Figure 12:**
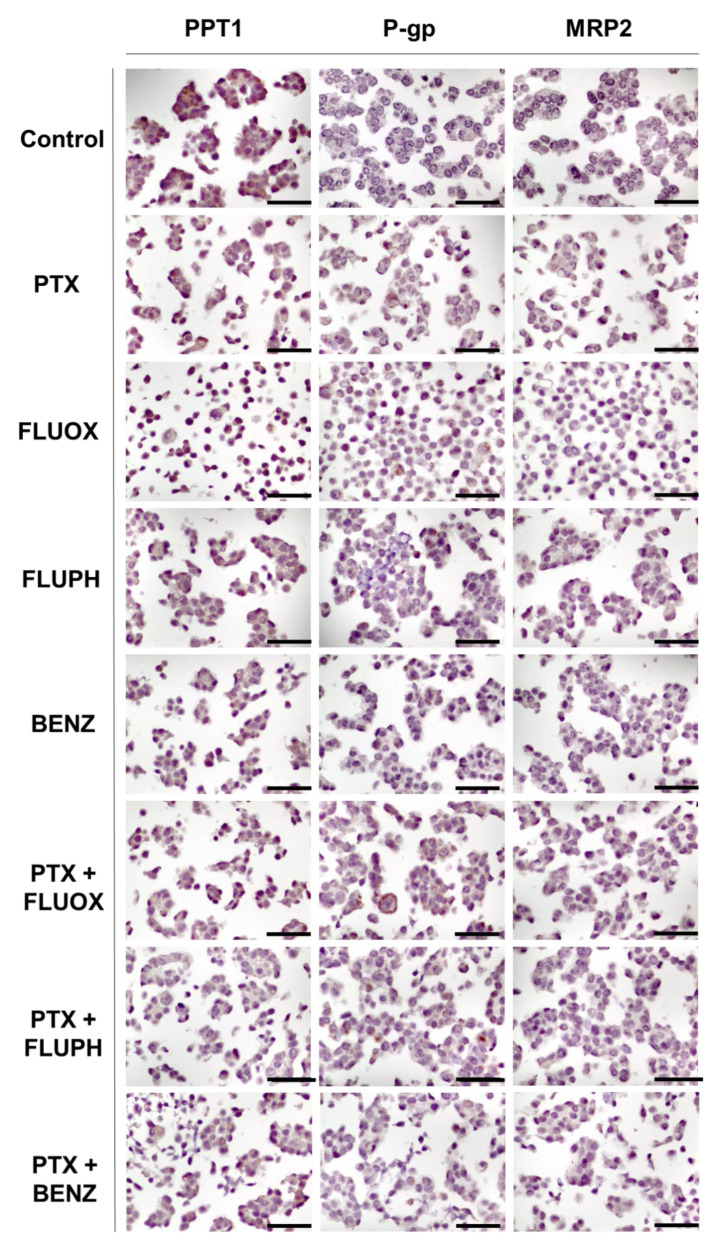
Representative images of PPT1, P-gp, and MRP2 immunohistochemistry (IHC) results of MCF-7 cells treated with CNS drugs (FLUOX, FLUPH, and BENZ), alone and in combination with PTX. All images are taken at magnification of 400×. Scale bar 50 µm.

Finally, it was evaluated the expression of PPT1, P-gp, and MRP-2 in MCF-7 cells treated with a combination of PTX plus CNS drugs FLUOX, FLUPH, and SERT (Figure 12). It was found low expression (11–25%) of PPT1 in MCF-7 cells treated with PTX and FLUPH alone (Figure 12). Treatment with FLUOX alone led to an increase in PPT1 expression to 76–100%, while treatment with BENZ resulted in only 1–10% of MCF-7 expressing this protein (Figure 12). Results of MCF-7 cells treated with PTX + FLUOX and PTX + BENZ demonstrated 11–25% of positive cells for this marker (Figure 12). Interestingly, the combination of PTX + FLUPH demonstrated the capacity to inhibit PPT1, with negative expression of this protein (Figure 12). Treatment with PTX, FLUPH, and BENZ alone resulted in 11–25% positive cells for PPT1 expression. Treatment with FLUOX revealed a higher expression (51–75%) of the protein (Figure 12). MCF-7 cells treated with PTX + BENZ revealed a similar expression of PPT1 protein to each drug alone (11–25%). The combination of PTX + FLUOX also demonstrated a reduction in the percentage of positive cells (26–50%) (Figure 12). On the other side, MCF-7 cells treated with PTX + FLUPH revealed an increased expression of PPT1 (26–50%), compared to both drugs alone (Figure 12).

It was found low expression of P-gp in MCF-7 cells treated with PTX, FLUPH, and BENZ alone (11–25%) (Figure 12). Treatment with FLUOX alone led to an increase in P-gp expression to 51–75%. Results of MCF-7 cells treated with PTX + BENZ demonstrated 11–25% of positive cells for this marker, similar to the treatment with each drug alone (Figure 12). On the other side, the combination of PTX + FLUOX demonstrated the ability to reduce P-gp expression (26–50%) (Figure 12). In the treatment of PTX + FLUPH, it was found an increase in P-gp expression compared to single drugs, with 26–50% positive cells (Figure 12). Results regarding MRP-2 expression demonstrated this protein is not expressed in these cells in any treatment (Figure 12). 

**Figure 13:**
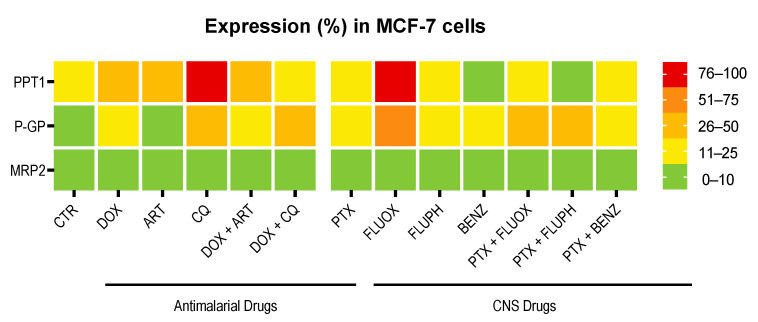
Heat map showing the percentage of positive cells for PPT1, P-gp, and MRP-2 for all the conditions tested. MCF-7 cells were treated with antineoplastic (DOX and PTX) and repurposed drugs (ART, CQ, FLUOX, FLUPH, and BENZ), both alone and in combination. The color key represents the percentage of positive cells for each marker.

The results regarding Ki67, cleaved-PARP, and NF-kB p65 expression in MCF-7 cells are summarized in Figure S6. Results about PPT1, P-gp, and MRP-2 expression in MCF-7 cells treated with antineoplastic and repurposed drugs are summarized in Figure 13. 

#### 3.2.3. HT-29 Cells Treated with 5-FU + CNS Drugs

Next, it was evaluated the expression of Ki67, cleaved-PARP, and NF-kB p65 markers in HT-29 cells treated with a combination of 5-FU plus the CNS drugs THIO and SERT (Figure S7). In HT-29 control cells, it was found an abundant nuclear expression (76–100%) of Ki67. Except for the treatment of HT-29 cells with 5-FU, all conditions demonstrated a similar percentage of Ki67 positive cells to the control (Figure S7). Treatment with the antineoplastic drug 5-FU induced a reduction in cell viability, as demonstrated by a decrease (26–50%) in Ki67 expression (Figure S7).

It was not found significant changes in cleaved-PARP and NF-kB p65 expression among treatments with single and combined drugs, with the expression of both markers being negative or <1% (Figure S7). 

**Figure 14:**
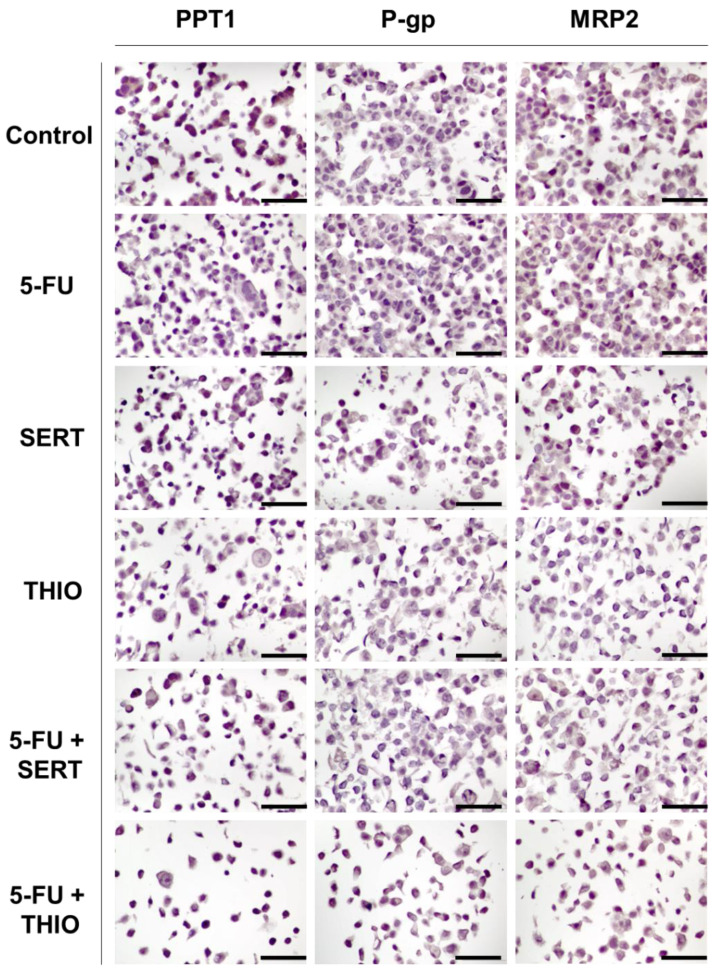
Representative IHC images for PPT1, P-gp, and MRP2 expression in HT-29 cells treated with antimalarial drugs (SERT and THIO), alone and in combination with 5-FU. All images are taken at a magnification of 400×. Scale bar 50 µm.

Regarding the expression of PPT1, P-gp, and MRP-2 in HT-29 cells treated with a combination of 5-FU plus CNS drugs THIO and SERT (Figure 14), we have found high (51–75%) expression of PPT1 in HT-29 control cells (Figure 14). Interestingly, treatments with single drugs and in combination seemed to inhibit this enzyme, with almost no HT-29 cells expressing this protein. Results regarding MRP-2 and P-gp expression demonstrated these proteins were not expressed in these cells in any treatment (Figure 14).

**Figure 15:**
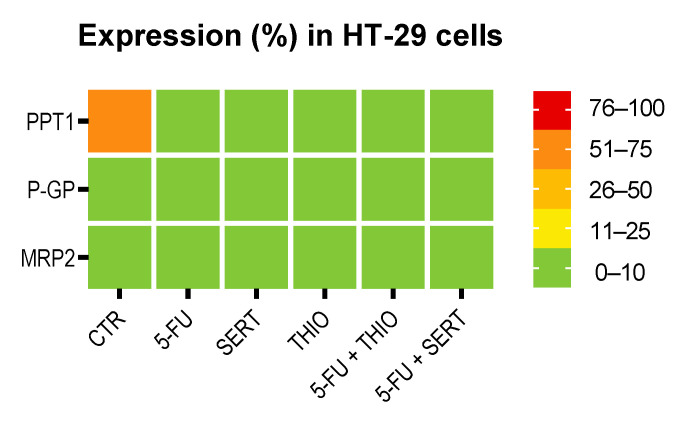
Heat map showing the percentage of positive cells for PPT1, P-gp, and MRP-2 for all the conditions tested. HT-29 cells were treated with antineoplastic (5-FU) and repurposed drugs (SERT and THIO), both alone and in combination. The color key represents the percentage of positive cells for each marker.

The results regarding Ki67, cleaved-PARP, and NF-kB p65 expression in HT-29 cells are summarized in Figure S8. Results about PPT1, P-gp, and MRP-2 expression in HT-29 cells treated with antineoplastic and repurposed drugs are summarized in Figure 15. 

**Figure 16:**
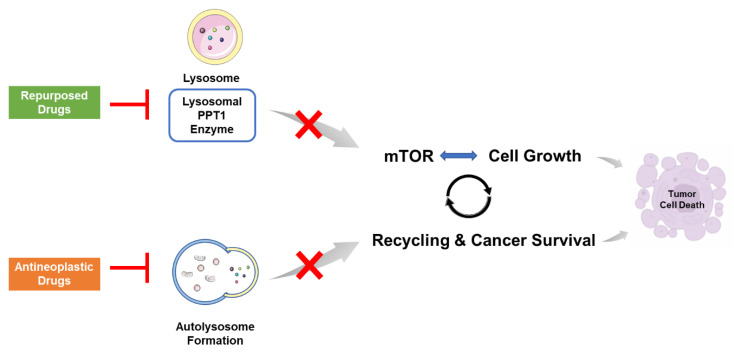
Proposed model for the action of combination therapies using repurposed and antineoplastic drugs in PPT1, a lysosomal enzyme. Antineoplastic drugs such as 5-FU, PTX, and DOX can inhibit autophagy, resulting in cell death. Nevertheless, some cells can be resistant to autophagy and survive, leading to cancer progression. Combination with repurposed drugs can generate a deeper response by inhibiting PPT1. This inhibition leads to mTOR inactivation, decreasing cellular protein synthesis and cell growth.

We have previously suggested in a prospective study that other antimalarial agents besides chloroquine, may interact with PPT1 and therefore inhibit the mTOR signaling and the autophagic flux [19]. In that study, our group hypothesized that PPT1 inhibition by antimalarial drugs could result in changes in the lysosomal metabolism that contribute to less accumulation of antineoplastic drugs in lysosomes, increasing the bioavailability of the antineoplastic agents. Indeed, several studies indicate that doxorubicin co-localizes with lysosomes and autophagosomes in breast cells resistant to doxorubicin [47]. Furthermore, it was found that inhibitors of lysosomal function such as chloroquine potentiate the anticancer effect of doxorubicin in hepatic cancer cells [48]. Moreover, it was also demonstrated that paclitaxel inhibits endosomal-lysosomal membrane trafficking, by inducing early accumulation of protein and endocytic markers in endosomes and later accumulation in a subdomain of lysosomes [49]. Other studies demonstrated that enhanced lysosomal function is responsible for paclitaxel resistance in cancer cells and that this drug resistance can be reverted using artesunate or other inhibitors of lysosomal function [50]. Similar findings were reported in relation to 5-FU, where the inhibition of the autophagy process, either by chloroquine or lysosomal photodamage, contributed to increased sensitivity of pancreatic cancer cells to 5-FU [51]. Here, we demonstrate that not only antimalarial drugs but also CNS drugs can target PPT1 and inhibit this protein. To explain the synergistic effect among antineoplastic and repurposed drugs in breast and colon cancer cells, we propose that (1) antineoplastic drugs such as 5-FU, PTX, and DOX inhibit autophagy, resulting in cell death; nevertheless, some cells resist autophagy and survive, leading to cancer progression (2) the repurposed drugs affect lysosomal metabolism, mainly through the inhibition of the PPT1 enzyme and consequently mTOR, helping decrease the accumulation of the antineoplastic drug in the lysosomes and consequently reducing the chance of drug resistance, enhancing their bioavailability, and increasing the antitumor effect of these drugs (Figure 16). 

## 4. Conclusions

We have successfully demonstrated that the combinations of antineoplastic drugs with antimalarial and CNS drugs have an acceptable toxicological profile. By studying their cytotoxic evaluation on MRC-5 cells, it was demonstrated these combinations are safe in lower doses, without significant cell viability reduction of MRC-5 fibroblast cells. It was also proved these combinations induce cytotoxicity for cancer cells, even in the concentration range that is compatible with non-tumoral cells. 

Our results also demonstrate that PPT1 might be involved in the mechanism of action of the aforementioned drug combinations and demonstrate that both antimalarial and CNS drugs in combination with chemotherapeutic agents can decrease the PPT1 expression in MCF-7 breast and HT-29 colon cell lines. These results suggest that PPT1 may be a target not only of antimalarial drugs, as already described in previous studies, but also of CNS drugs, both alone and in combination. We propose a model for the action of these combination therapies in breast and colon cancer in which the antineoplastic drugs inhibit autophagy, resulting in cell death, with the simultaneous targeting of PPT1 by the repurposed drugs, leading to mTOR inactivation and consequently decreasing the cellular protein synthesis and cancer cell growth. Nevertheless, more studies are necessary to further explore the complete mechanisms of action behind these drug combinations. 

Taken together, we demonstrate that antimalarial and CNS drugs can be safely used in combination regimens with chemotherapeutic agents and support further studies of these drug combinations in more complex systems such as animal models. 

## Figures and Tables

**Table 1:** Half-maximal inhibitory concentration (IC_50_) of reference and repurposed drugs in Michigan Cancer Foundation-7 (MCF-7, breast cancer) and human colorectal adenocarcinoma (HT-29) cells.

Drug	HT-29	MCF-7
	IC_50_ (µM)	IC_50_ (µM)
DOX	N.D.	0.17
PTX	N.D	2.78 (nM)
5-FU	3	N.D.
FLUOX	6.12	7.78
BENZ	18.23	21.71
THIO	4.26	5.72
SERT	2.45	2.22
FLUPH	1.86	2.68
ART	17.88	11.60
CQ	32.13	63.98 *

N.D.—Not determined. * IC_50_ obtained from the literature [28].

**Table 2:** Selectivity index (SI) and IC_50_ values of doxorubicin (DOX), paclitaxel (PTX), and 5-fluorouracil (5-FU) in non-tumoral (MRC-5) and tumoral (MCF-7 and HT-29) cells.

Drug	IC_50_ (Non-Tumoral Cells)	IC_50_ (Tumoral Cells)	SI
DOX	0.43 µM	0.17 µM (MCF-7)	2.53
PTX	2.61 nM	2.78 nM (MCF-7)	0.94
5-FU	5.78 µM	3.00 µM (HT-29)	1.93

**Table 3:** SI and IC_50_ values of DOX and chloroquine (CQ) in non-tumoral (MRC-5) and tumoral (MCF-7) cells.

Drug	IC_50_ (Non-Tumoral Cells)	IC_50_ (Tumoral Cells)	SI
ART	14.70 µM	11.60 µM	1.27
CQ	14.85 µM	63.98 µM	0.23

**Table 4:** SI and IC_50_ values of fluoxetine (FLUOX), fluphenazine (FLUPH), benztropine (BENZ), thioridazine (THIO), and sertraline (SERT) in non-tumoral (MRC-5) and tumoral (MCF-7 and HT-29) cells.

Drug	IC_50_ (Non-Tumoral Cells)	IC_50_ (Tumoral Cells)	SI
FLUOX	16.59 µM	7.78 µM (MCF-7)	2.13
FLUPH	6.87 µM	2.68 µM (MCF-7)	2.56
BENZ	19.91 µM	21.71 µM (MCF-7)	0.92
THIO	5.46 µM	4.26 µM (HT-29)	1.28
SERT	5.20 µM	2.45 µM (HT-29)	2.12

## Data Availability

Not applicable.

## Supplementary Materials

The following supporting information can be downloaded at: https://www.mdpi.com/article/10.3390/biom12101490/s1, Table S1: List of antibodies used for immunohistochemistry studies. EDTA—ethylenediamine tetraacetic acid; mAb—monoclonal antibody; pAb—polyclonal antibody; RT—room temperature.; Figure S1: Representative immunohistochemistry (IHC) images for (A) PPT1; (B) P-gp and (C) MRP2 in a positive control (liver). All the images are taken at magnification of 200×. Scale bar 100 µm.; Figure S2: Representative IHC images for Ki67, cleaved-PARP, and NF-kB p65 expression in MCF-7 cells treated with antimalarial drugs (ART and CQ), alone and in combination with DOX. All images are taken at magnification of 400×. Scale bar 50 µm.; Figure S3: Representative IHC images for Ki67, cleaved-PARP, and NF-kB p65 expression in MCF-7 cells treated with CNS drugs (FLUOX, FLUPH, and BENZ), alone and in combination with PTX. All images are taken at magnification of 400×. Scale bar 50 µm.; Figure S4: Heat map showing the percentage of positive cells for Ki67, cleaved-PARP, and NF-kB p65 for all the conditions tested. MCF-7 cells were treated with antineoplastic (DOX and PTX) and repurposed drugs (ART, CQ, FLUOX, FLUPH, and BENZ), both alone and in combination. The color key represents the percentage of positive cells for each marker.; Figure S5: Representative IHC images for Ki67, cleaved-PARP, and NF-kB p65 expression in HT-29 cells treated with antimalarial drugs (SERT and THIO), alone and in combination with 5-FU. All images are taken at magnification of 400×. Scale bar 50 µm.; Figure S6: Heat map showing the percentage of positive cells for Ki67, cleaved-PARP, and NF-kB p65 for all the conditions tested. HT-29 cells were treated with antineoplastic (5-FU) and repurposed drugs (SERT and THIO), both alone and in combination. The color key represents the percentage of positive cells for each marker.
