# Supplementary material for: Combination of Antimalarial and CNS Drugs with Antineoplastic Agents in MCF-7 Breast and HT-29 Colon Cancer Cells: Biosafety Evaluation and Mechanism of Action
Source: Biomolecules. 2022 Oct 16;12(10):1490. doi: 10.3390/biom12101490 (PMC9599492; doi:10.3390/biom12101490)
Supplement: Supplementary file 1 [file biomolecules-12-01490-s001.zip › biomolecules-1924878-supplementary.pdf]

biomolecules

Combination of Antimalarial and CNS Drugs with Antineoplastic Agents in MCF-7 Breast and HT-29 Colon Cancer Cells: Biosafety Evaluation and Mechanism of Action

Diana Duarte [1,2], Mariana Nunes [3,4], Sara Ricardo [3,5,6] and Nuno Vale [1,2,7,*]

1 OncoPharma Research Group, Center for Health Technology and Services Research (CINTESIS), Rua Doutor Plácido da Costa, 4200-450 Porto, Portugal
2 Faculty of Pharmacy, University of Porto, Rua Jorge Viterbo Ferreira, 228, 4050-313 Porto, Portugal
3 CINTESIS@RISE, Faculty of Medicine, University of Porto, Alameda Professor Hernâni Monteiro, 4200-319 Porto, Portugal
4 Differentiation and Cancer Group, Institute for Research and Innovation in Health (i3S), University of Porto/Institute of Molecular Pathology and Immunology, University of Porto (IPATIMUP), Rua Alfredo Allen 208, 4200-135 Porto, Portugal
5 Institute of Biomedical Sciences Abel Salazar (ICBAS), University of Porto, Rua Jorge Viterbo Ferreira, 228, 4050-313 Porto, Portugal
6 Toxicology Research Unit (TOXRUN), University Institute of Health Sciences, Polytechnic and University Cooperative (CESPU), Rua Central de Gandra, 1317, 4585-116 Gandra, Portugal
7 Department of Community Medicine, Health Information and Decision (MEDCIDS), Faculty of Medicine, University of Porto, Rua Doutor Plácido da Costa, 4200-450 Porto, Portugal
* Correspondence: nunovale@med.up.pt; Tel.: +351-220426537

Supplementary Material

Table S1. List of antibodies used for immunohistochemistry studies. EDTA—ethylenediamine tetraacetic acid; mAb—monoclonal antibody; pAb—policlonal antibody; RT—room temperature.

Antigen	Clone	Reference	Origin	Animal origin	Antigen retrieval buffer	Dilution	Incubation conditions	Localization
MRP2	4C9.2	MABN1545	Sigma-Aldrich	Mouse mAb	EDTA	1:50	60′ RT	Membrane
NF-kB p65	C-20	SC-372	Santa Cruz Biotechnology	Rabbit pAb	Citrate at pH 6	1:600		Cytoplasm
Cleaved-PARP	Asp214 D64E10	5625	Cell Signalling Technology	Rabbit mAb	Citrate at pH 6	1:50		Nuclear
PPT1	-	PA5-79860	ThermoFisher Scientific	Rabbit pAb	Citrate at pH 6	1:500		Cytoplasm
Ki67	SP6	MA5-14530	ThermoFisher Scientific	Rabbit mAb	Citrate at pH 6	1:100		Nuclear
P-gp	C219	MA1-26528	ThermoFisher Scientific	Mouse mAb	EDTA	1:25		Membrane

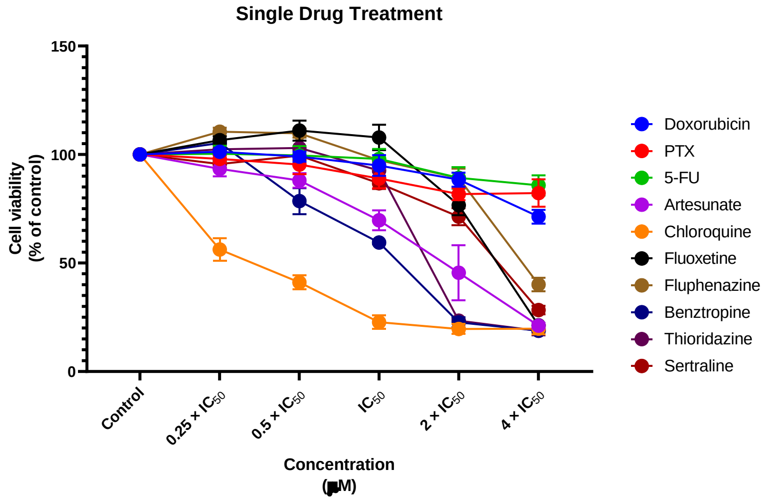

Figure S1. Comparison of the cytotoxic effect after single drug treatments with antineoplastic, antimalarial, and CNS drugs in MRC-5 human normal lung fibroblast cells. Results are represented as the percentage of control and represent means ± SEM. Each experiment was done three times independently ($n = 3$).

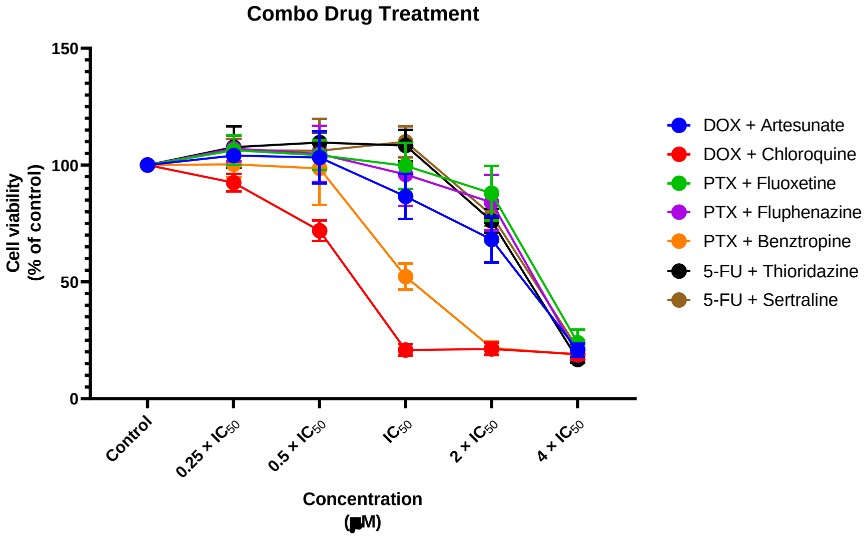

Figure S2. Comparison of the cytotoxic effect after combination treatments with antineoplastic and repurposed drugs in MRC-5 human normal lung fibroblast cells. Results are represented as the percentage of control and represent means ± SEM. Each experiment was done three times independently ($n = 3$).

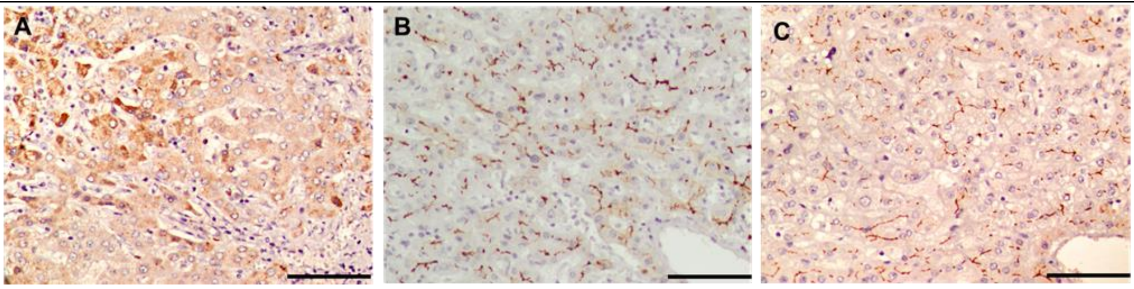

Figure S3. Representative immunohistochemistry (IHC) images for (A) PPT1; (B) P-gp and (C) MRP2 in a positive control (liver). All the images are taken at magnification of 200x. Scale bar 100 μm.

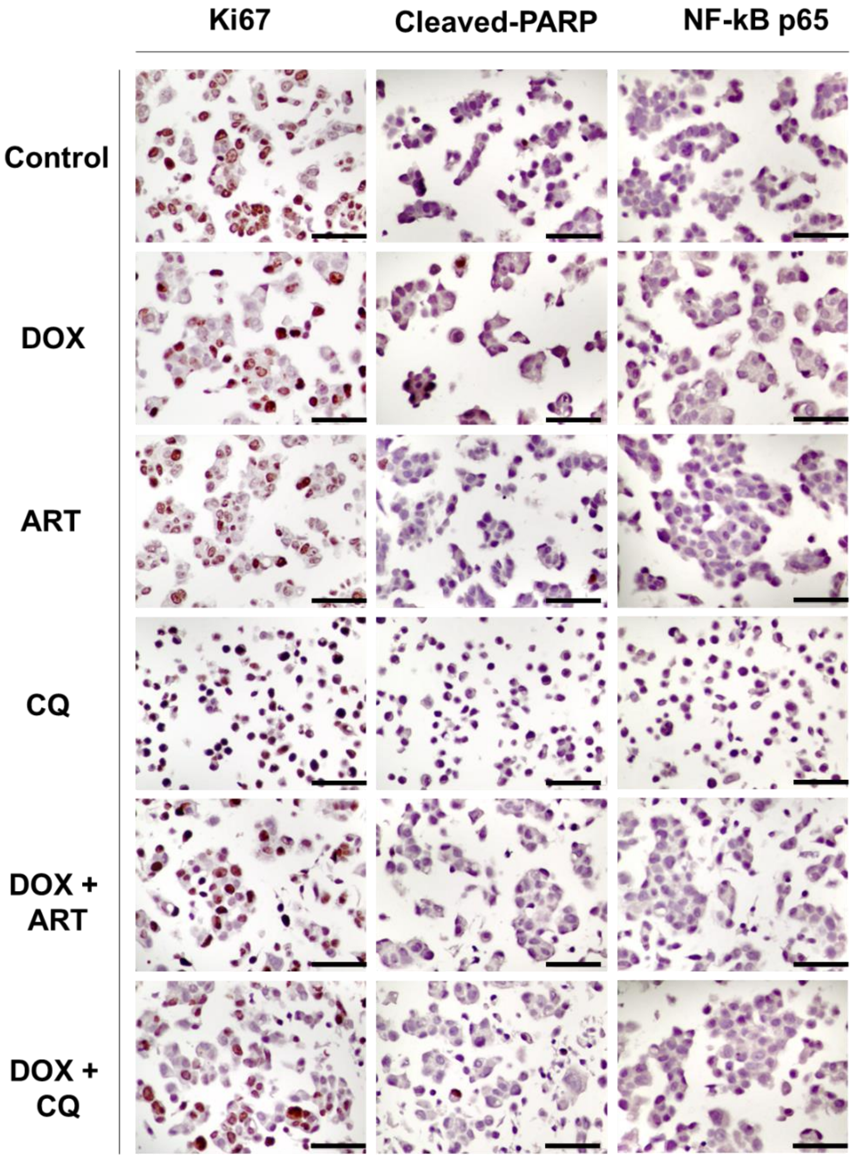

Figure S4. Representative IHC images for Ki67, cleaved-PARP, and NF-kB p65 expression in MCF-7 cells treated with antimalarial drugs (ART and CQ), alone and in combination with DOX. All images are taken at magnification of 400x. Scale bar 50 μm.

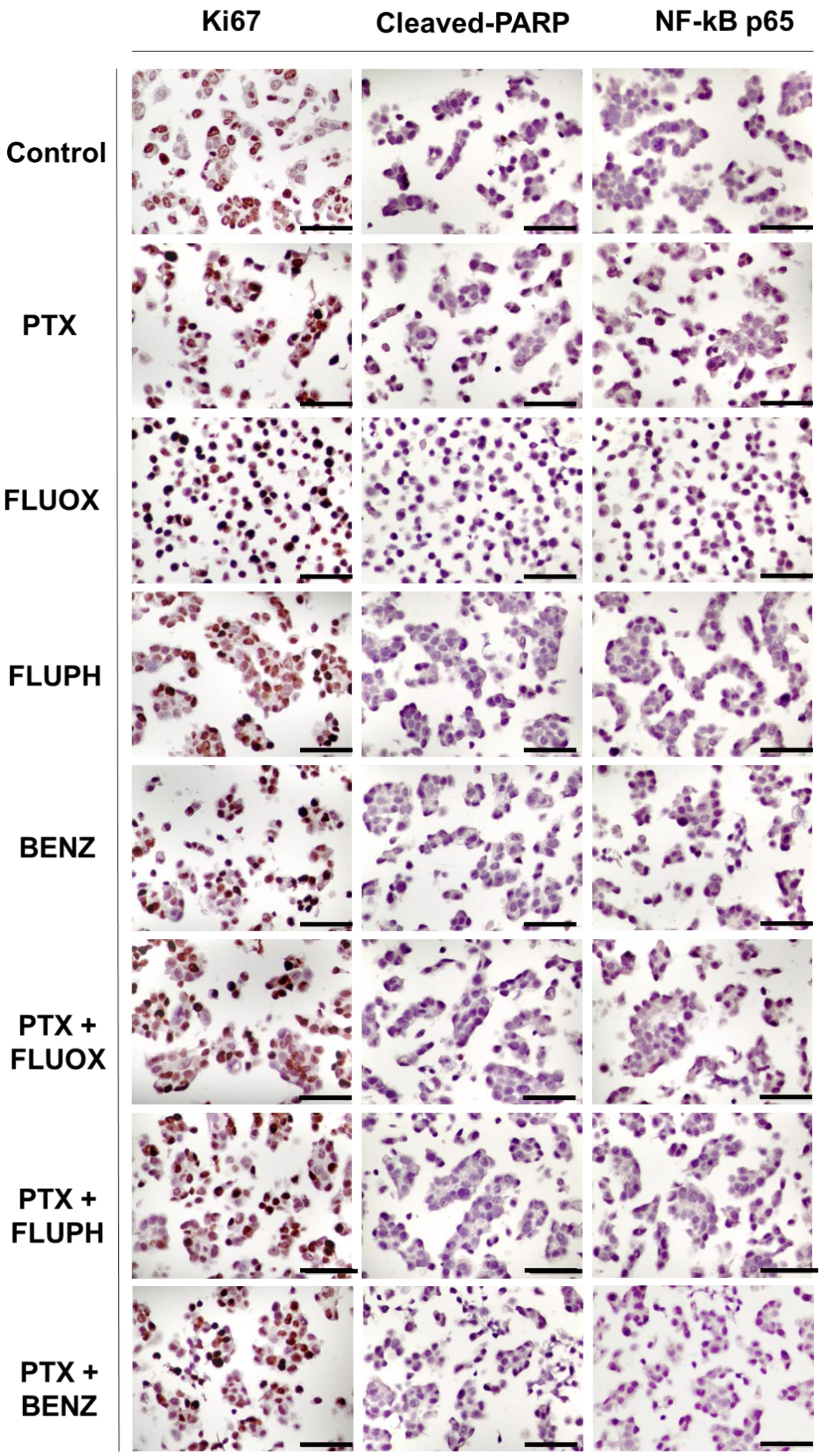

Figure S5. Representative IHC images for Ki67, cleaved-PARP, and NF-kB p65 expression in MCF-7 cells treated with CNS drugs (FLUOX, FLUPH, and BENZ), alone and in combination with PTX. All images are taken at magnification of 400×. Scale bar 50 µm.

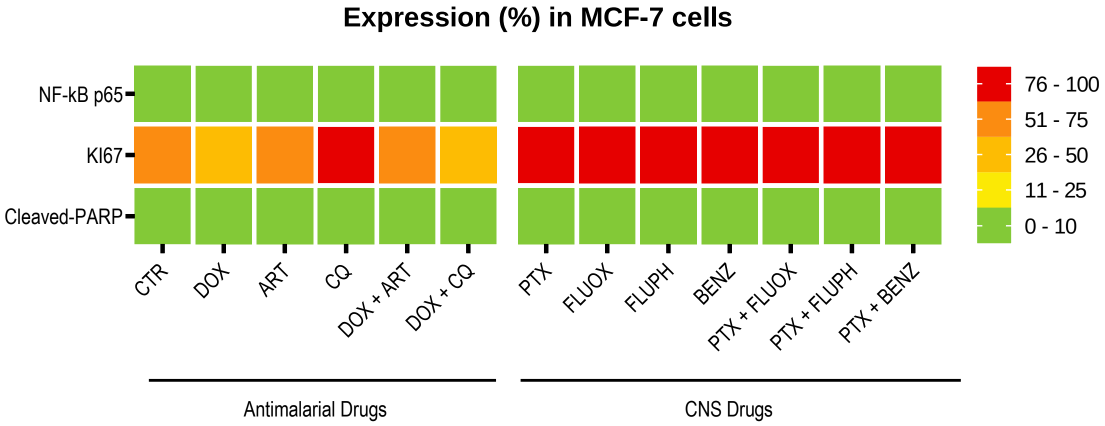

Figure S6. Heat map showing the percentage of positive cells for Ki67, cleaved-PARP and NF-kB p65 for all the conditions tested. MCF-7 cells were treated with antineoplastic (DOX and PTX) and repurposed drugs (ART, CQ, FLUOX, FLUPH, and BENZ), both alone and in combination. The color key represents the percentage of positive cells for each marker.

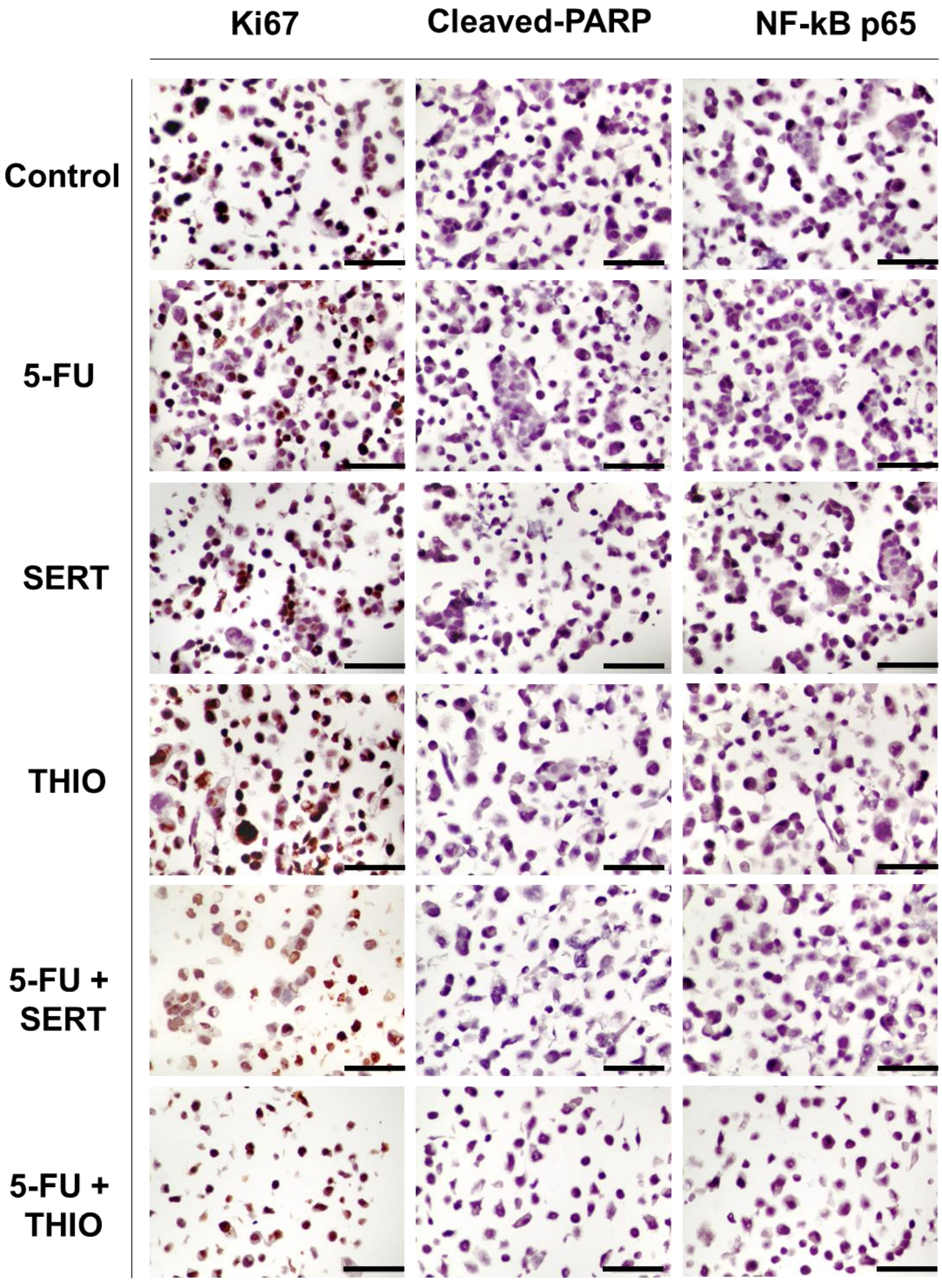

Figure S7. Representative IHC images for Ki67, cleaved-PARP, and NF-kB p65 expression in HT-29 cells treated with antimalarial drugs (SERT and THIO), alone and in combination with 5-FU. All images are taken at magnification of 400x. Scale bar 50 μm.

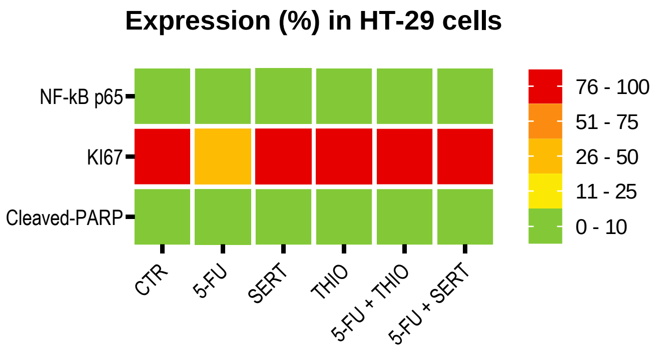

Figure S8. Heat map showing the percentage of positive cells for Ki67, cleaved-PARP and NF-kB p65 for all the conditions tested. HT-29 cells were treated with antineoplastic (5-FU) and repurposed drugs (SERT and THIO), both alone and in combination. The color key represents the percentage of positive cells for each marker.
